# Examining the mental health services among people with mental disorders: a literature review

**DOI:** 10.1186/s12888-024-05965-z

**Published:** 2024-08-20

**Authors:** Yunqi Gao, Richard Burns, Liana Leach, Miranda R. Chilver, Peter Butterworth

**Affiliations:** 1 The National Centre for Epidemiology and Population Health, Australian National University, Canberra, Australia; 2 School of Psychology, Deakin University, Melbourne, Australia; 3 Department of Health, Economics, Wellbeing and Society, Australian National University, Canberra, Australia

**Keywords:** Health services, Service use, Stigma, Mental disorder, Systematic review

## Abstract

**Background:**

Mental disorders are a significant contributor to disease burden. However, there is a large treatment gap for common mental disorders worldwide. This systematic review summarizes the factors associated with mental health service use.

**Methods:**

PubMed, Scopus, and the Web of Science were searched for articles describing the predictors of and barriers to mental health service use among people with mental disorders from January 2012 to August 2023. The initial search yielded 3230 articles, 2366 remained after removing duplicates, and 237 studies remained after the title and abstract screening. In total, 40 studies met the inclusion and exclusion criteria.

**Results:**

Middle-aged participants, females, Caucasian ethnicity, and higher household income were more likely to access mental health services. The use of services was also associated with the severity of mental symptoms. The association between employment, marital status, and mental health services was inconclusive due to limited studies. High financial costs, lack of transportation, and scarcity of mental health services were structural factors found to be associated with lower rates of mental health service use. Attitudinal barriers, mental health stigma, and cultural beliefs also contributed to the lower rates of mental health service use.

**Conclusion:**

This systematic review found that several socio-demographic characteristics were strongly associated with using mental health services. Policymakers and those providing mental health services can use this information to better understand and respond to inequalities in mental health service use and improve access to mental health treatment.

**Supplementary Information:**

The online version contains supplementary material available at 10.1186/s12888-024-05965-z.

## Introduction

 Mental disorders such as depression and anxiety are prevalent, with nationally representative studies showing that one-fifth of Australians experience a mental disorder each year [5]. More recent estimates derived from a similar survey during the period of the COVID-19 pandemic were 21.5% [11]. Mental illness can reduce the quality of life, and increase the likelihood of communicable and non-communicable diseases [116, 137], and is among the costliest burdens in developed countries [22, 34, 80]. The National Mental Health Commission [96] stated that the annual cost of mental ill-health in Australia was around $4000 per person or $60 billion. The Global Burden of Diseases, Injuries, and Risk Factors Study (GBD) 2019 reported that mental disorders rank the seventh leading cause of disability-adjusted life years and the second leading cause of years lived with disability [48]. Helliwell et al. [56] indicated that chronic mental illness was a key determinant of unhappiness, and it triggered more pain than physical illness. Mental health issues can have a spillover effect on all areas of life, poor mental health conditions might lead to lower educational achievements and work performance, substance abuse, and violence [102]. In Australia, despite considerable additional investment in the provision of mental health services research suggests that the rate of psychological distress at the population level has been increasing [38], this has been argued to reflect that people who most need mental health treatment are not accessing services. Insufficient numbers of mental health services and mental healthcare professionals and inadequate health literacy have been reported as the pivotal determinants of poor mental health [18]. Previous studies have reported large treatment gaps in mental health services; finding only 42–44% of individuals with mental illness seek help from any medical or professional service provider [85, 112] and this active proportion was much lower in low and middle-income countries [32, 114, 130].

Several studies have investigated factors associated with high and low rates of mental health service use and identified potential barriers to accessing mental health service use. Demographic, social, and structural factors have been associated with low rates of mental health service use. Structural barriers include the availability of mental health services and high treatment costs, social barriers to treatment access include stigma around mental health [125], fear of being perceived as weak or stigmatized [79], lack of awareness of mental disorders, and cultural stigma [17].

Existing studies that have systematically reviewed and evaluated the literature examining mental health service use have largely been constrained to specific population groups such as military service members [63] and immigrants [33], children and adolescents [35], young adults [76], and help-seeking among Filipinos in the Philippines [93]. These systematic reviews emphasize mental health service use by specific age groups or sub-groups, and the findings might not represent the patterns and barriers to mental health service use in the general population. One paper has reviewed mental health service use in the general adult population. Roberts et al. [112] found that need factors (e.g. health status, disability, duration of symptoms) were the strongest determinants of health service use for those with mental disorders.

The study results from Roberts et al. [112] were retrieved in 2016, and the current study seeks to build on this prior review with more recent research data by identifying publications since 2012 on mental health service use with a focus on high-income countries. This is in the context of ongoing community discussion and reform of the design and delivery of mental health services in Australia [140], and the need for current evidence to inform this discussion in Australia and other high-income countries. This systematic review aims to investigate factors associated with mental health service use among people with mental disorders and summarize the major barriers to mental health treatment. The specific objectives are (1) to identify factors associated with mental health service use among people with mental disorders in high-income countries, and (2) to identify commonly reported barriers to mental health service use.

## Methodology

### Selection procedures

Our review adhered to PRISMA guidelines to present the results. We utilized PubMed, Scopus, and the Web of Science to search for articles describing the facilitators and barriers to mental health service use among people with mental illness from January 2012 up to August 2023. There were no specific factors that were of interest as part of conducting this systematic review, instead, the review had a broad focus intending to identify factors shown to be associated with mental health service use in the recent literature. The keywords used in our search of electronic databases were related to mental disorders and mental health service use. The full search terms and strategies were shown in Supplementary Table 1. We uploaded the search results to Covidence for deduplication and screening. After eliminating duplicates, the first author retrieved the title abstract and full-text articles for all eligible papers. Then each title and abstract were screened by two independent reviewers, to select those that would progress to full-text review. Subsequently, the two reviewers screened the full text of all the selected papers and conducted the data extraction for those that met the eligibility criteria. There were discrepancies in 12% of the papers reviewed, and all conflicts were resolved through discussion and agreed on by at least three authors.

### Selection criteria

#### Inclusion and exclusion criteria

In this systematic review, the scope was restricted to studies that draw samples from the general population, and the participants were either diagnosed with mental disorders or screened positive using a standardized scale. Case-control studies and cohort studies were considered for inclusion. The applied inclusion and exclusion criteria are listed in Table 1.

**Table 1:** Inclusion and exclusion criteria

Inclusion criteria	Exclusion criteria
Participants are sampled from a known population (e.g. area/ location).	Randomized control trials.
Participants met diagnostic criteria or screened positive for mental disorders.	Studies report help-seeking among people with chronic diseases (unless also specifies mental health problems).
The study collects data on study participants’ access to mental health services.	Studies focus on certain sub-populations (unless it contained a comparison group with the general population).
Studies conducted in high-income countries, use World Bank income groups [141] to identify high-income countries.	Studies that only consider general health-service use (e.g. primary care/ general GP consultations).
There are no restrictions on age, gender, occupation, and hobbies.	Studies excluded if restricted to specific ethnic culture or linguistic groups.
Must be full-text peer-reviewed articles published in journals or book chapters.	Conference papers, editorials, systematic reviews, meta-analyses, and other forms of literature review.
Papers published in English.	Studies with less than 100 participants.

### Data extraction

After the full-text screening, details from all eligible studies were extracted by field into a data extraction table with thematic headings. The descriptive data includes the study title, author, publication year, geographic location, sample size, population details (gender, age), type of study design, mental disorder type (medical diagnosis or using scales) and quality grade (e.g. good, fair, and poor).

### Quality assessment

The Newcastle Ottawa Scale [136] was used to evaluate the study quality for all eligible papers. We assessed the cross-sectional and cohort studies using separate assessment forms and graded each study as good, fair, or poor. The quality grade for each study was included in the data extraction table. The first author conducted the quality assessment using the Newcastle Ottawa Scale for cohort studies and the adapted scale for cross-sectional studies.

## Results

**Fig. 1:**
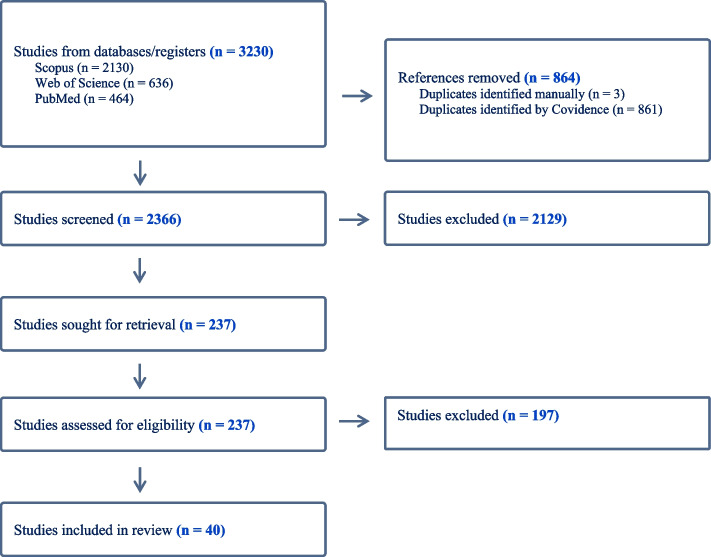
Flowchart for selections of studies

The search process is summarized in Fig. 1. The initial search from PubMed, Scopus, and the Web of Science yielded 3230 articles: 2366 remained after removing duplicates; 2129 studies were considered not relevant; and 237 studies remained following title and abstract screening. In total, 40 studies met the inclusion criteria. Of these, four were cohort studies while thirty-six were cross-sectional studies. Ten studies (25.0%) were conducted in Canada, and nine (22.5%) were from the United States. Three studies used data from Germany (7.5%). Two studies each reported data from Australia, Denmark, Sweden, Singapore, or South Korea (5.0% of studies for each country). A single study was included with data from either the United Kingdom, Italy, Israel, Portugal, Switzerland, Chile, New Zealand, or reported pooled multinational data from six European countries (each country/ study representing 2.5% of the total sample of studies) (Table 2).

**Table 2:** Basic study information

Author & Published Year	Country	Study Design	Sample Size	Data Source	Reference Period
Abramovich et al., 2020 [2]	Canada	Cross-sectional	12,510	Institute for Clinical Evaluative Sciences data	12-month
Caron et al., 2012 [21]	Canada	Cross-sectional	2,433	Quantitative survey, aged 15 to 65 residents in the City of Montreal.	12-month
Chiu et al., 2018 [24]	Canada	Cohort	238,392	Statistics Canada’s cross-sectional Canadian Community Health Survey	12-month
Choi et al., 2014 [26]	United States	Cross-sectional	96,966	National Survey of Drug Use and Health	12-month
Chong et al., 2012 [27]	Singapore	Cross-sectional	6,616	Singapore Mental Health Study	12-month
Chow & Mulder, 2017 [28]	New Zealand	Cross-sectional	229,874	Program for the Integration of Mental Health Data	5-year rate of service use
De Luca et al., 2015 [30]	United States	Cross-sectional	8,563	Texas Behavioral Risk Factor Surveillance System	Lifetime treatment
Durbin et al., 2015 [31]	Canada	Cross-sectional	912,114	Citizenship and Immigration Canada, the Registered Persons Database	Lifetime treatment
Elbogen et al., 2013 [37]	United States	Cross-sectional	1,102	National Post-Deployment Adjustment Survey	12-month
Erlangse et al., 2017 [39]	Denmark	Cross-sectional	7,006,898	Use personal identification number linked to Danish social and health data	Lifetime treatment
Fleury et al., 2012 [21]	Canada	Cross-sectional	406	Quantitative survey, aged 15 to 65 residents in the south-western sector of Montreal	12-month
Forslund et al., 2020 [43]	Sweden	Cohort	1,472,348 in 2007. 1,758,337 in 2017	Administrative health care data register of the Stockholm Region	7 years rate of service use
Gazibara et al., 2021 [45]	Denmark	Cross-sectional	63,684	Use personal identification number linked to Danish social and health data	24-month before & after decedent’s death
Glasheen et al., 2019 [46]	United States	Cross-sectional	229,600	2010 to 2014 National Surveys on Drug Use and Health	12-month
Goncalves et al., 2014 [47]	Australia	Cross-sectional	3,178	Australian National Survey of Mental Health and Wellbeing	12-month
Harber-Aschan et al., 2019 [54]	United Kingdom	Cross-sectional	1,052	Southeast London Community Health Study	12-month
Huynh et al., 2016 [55]	Canada	Cross-sectional	3,295	Montreal Longitudinal Catchment Area Study	12-month
Islam et al., 2017 [66]	Canada	Cross-sectional	3,995	Canadian Community Health Survey	12-month
Jacobi & Groß, 2014 [67]	Germany	Cross-sectional	5,317	German Health Interview and Examination Survey, Mental Health Module	12-month
Kaul et al., 2017 [68]	United States	Cross-sectional	1,750	National Health Interview Survey	12-month
Lipson et al., 2019 [87]	United States	Cohort	155,026	Healthy Minds Study	12-month
Mack et al., 2014 [90]	Germany	Cross-sectional	4,483	German Health Interview and Examination Survey for Adults	12-month
Park et al., 2012 [100]	South Korea	Cross-sectional	362	Korean Epidemiologic Catchment Area	Lifetime treatment
Pelletier et al., 2017 [104]	Canada	Cross-sectional	23,416	2012 Canadian Community Health Survey—Mental Health	12-month
Reaume et al., 2021 [110]	Canada	Cross-sectional	5,630	Canadian Community Health Survey-Mental Health (2012)	12-month
Reavley et al., 2020 [111]	Australia	Cross-sectional	655	Quantitative survey involving computer-assisted telephone interviews, members of the Australian community aged 18+	12-month
Shafie et al., 2021 [119]	Singapore	Cross-sectional	6,126	Singapore Mental Health Study 2016	12-month
Spinogati et al., 2015 [123]	Italy	Cross-sectional	8,755	Regional mental health information system	12-month
Volkert et al., 2018 [128]	Six European countries	Cross-sectional	3,142	Mental Disorders in the Elderly based on the International Classification of Functioning, Disability and Health Model	12-month
Wang et al., 2019 [129]	United States	Cross-sectional	37,224	2012 National Surveys on Drug Use and Health	12-month
Werlen et al., 2020 [132]	Switzerland	Cross-sectional	3,840	Swiss Youth Epidemiological Study on Mental Health	12-month
Whitley et al., 2017 [134]	Canada	Cohort	2,433	Epidemiological Catchment Area Study of Montreal South-West	12-month
Yang et al., 2020 [139]	United States	Cross-sectional	116,525	National Survey on Drug Use and Health	12-month
Rim et al., 2023 [105]	South Korea	Cross-sectional	5,511	The National Mental Health Survey of Korea 2021	12-month
Chaudhry et al., 2022 [23]	United States	Cross-sectional	172,209	National Health Interview Survey	12-month
Reich et al., 2023 [103]	Germany	Cross-sectional	1,180	Behavior and Mind Health study	Lifetime treatment
Errazuriz et al., 2023 [40]	Chile	Cross-sectional	1,264	Quantitative house- hold mental health survey of first-generation adult Peruvian immigrants with matched Chilean non-immigrants, aged 18-64 years	Past 3-month
Silvia et al., 2022 [120]	Portugal	Cross-sectional	809	2009 National Mental Health Survey	12-month
Björkenstam et al., 2022 [10]	Sweden	Cross-sectional	746,688	Longitudinal Integration Database for Health Insurance and Labor Market Studies, Longitudinal Database for Integration Studies, National Patient Register	Lifetime treatment
Blasbalg et al., 2023 [13]	Israel	Cross-sectional	928,044	Clalit Health Services	12-month

### Study characteristics

As shown in Tables 2, 3 and 4, the sample size of studies varies; a cross-sectional study from Canada had the largest sample which contained over seven million participants [39], while the smallest sample size was 362 [100]. Sixteen studies (40.0%) used DSM-IV diagnoses [4] to measure mental disorders, twelve studies (30.0%) applied the International Classification of Disease [138], and six studies used (15.0%) the Kessler Psychological Distress Scale [69]. Only three studies (7.5%) had a hospital diagnosis of mental disorders, while three studies (7.5%) used the Patient Health Questionnaire [72] to define mental disorders.

**Table 3:** Mental disorder and mental health service type

Author & published year	Mental disorder type	Medical diagnosis or scales (measurement)	Mental health service type
Abramovich et al., 2020 [2]	Common mental disorder	Research diagnostic interview: ICD-10 diagnoses from administrative databases	Mental health and psychiatrist visit, mental health-related hospitalization
Caron et al., 2012 [21]	Anxiety disorder, depressive disorder, substance-related disorder	Screening instrument/ scale: K10	Any mental health professional, hospitalization, support groups
Chiu et al., 2018 [24]	Common mental disorder	Survey questions based on diagnostic criteria	Any mental health professional
Choi et al., 2014 [26]	Anxiety disorder, depressive disorder, substance-related disorder, serious suicidal ideation	Survey questions based on diagnostic criteria: DSM-IV	Any mental health professional, support groups, spiritual or religious advisors, healer
Chong et al., 2012 [27]	Anxiety disorders, mood disorders and alcohol abuse disorders	Research diagnostic interview: ICD-10 diagnoses from administrative databases	Any mental health professional, support groups, spiritual or religious advisors, healer
Chow & Mulder, 2017 [28]	Anxiety disorder, major depressive disorder, bipolar and substance use disorder	Research diagnostic interview: ICD-10 diagnoses from administrative databases	Any mental health professional
De Luca et al., 2015 [30]	Common mental disorder (unspecified)	Survey questions based on diagnostic criteria: DSM-IV	Any mental health professional
Durbin et al., 2015 [31]	Common mental disorder (unspecified)	Clinical diagnosis: administrative data on primary care, ED and hospital admissions	Any mental health professional, hospitalization
Elbogen et al., 2013 [37]	Major depressive disorder, PTSD, alcohol use disorder	Screening instrument/ scale: Davidson Trauma Scale (DTS), Patient Health Questionnaire–9 (PHQ-9), and Alcohol Use Disorder Identification Test	Any mental health professional
Erlangse et al., 2017 [39]	Anxiety disorder, major depressive disorder, bipolar and substance use disorder, PTSD, alcohol use disorders, drug use disorder, deliberate self-harm	Research diagnostic interview: ICD-8 and ICD-10 diagnoses from administrative databases	Any mental health professional, hospitalization
Fleury et al., 2012 [21]	Major depressive disorder, PTSD, alcohol and drug use disorder	Research diagnostic interview: ICD-10 diagnoses from administrative databases	Any mental health professional, hospitalization, support groups
Forslund et al., 2020 [43]	Anxiety disorder, depressive disorder, substance-related disorder, developmental disorders, eating disorders	Research diagnostic interview: ICD-10 diagnoses from administrative databases	Psychiatric medication, psychological therapy
Gazibara et al., 2021 [45]	Anxiety disorder, depression, mood disorder, alcohol use disorder	Clinical diagnosis: registry data	Inpatient mental health treatment, outpatient mental health treatment
Glasheen et al., 2019 [46]	Common mental disorder	Survey questions based on diagnostic criteria: DSM-IV	Any mental health professional
Goncalves et al., 2014 [47]	Anxiety disorders, mood disorders and substance abuse disorders	Survey questions based on diagnostic criteria: DSM-IV	Any mental health professional
Harber-Aschan et al., 2019 [54]	Common mental disorder	Research diagnostic interview: ICD-10 diagnoses from administrative databases	Any mental health professional
Huynh et al., 2016 [55]	Anxiety disorder, depression, mood disorder, alcohol and drug use disorder	Research diagnostic interview: ICD-10 diagnoses from administrative databases	Any mental health professional, hospitalization, support groups
Islam et al., 2017 [66]	Common mental disorder (unspecified)	Survey questions based on diagnostic criteria: DSM-IV	Any mental health professional, support groups
Jacobi & Groß, 2014 [67]	Common mental disorder	Research diagnostic interview: ICD-10 diagnoses from administrative databases	Any mental health professional
Kaul et al., 2017 [68]	Common mental disorder	Screening instrument/ scale: K6	Any mental health professional
Lipson et al., 2019 [87]	Major depressive disorder suicidal ideation	Screening instrument/ scale: Patient Health Questionnaire–2 (PHQ-2)	Any mental health professional
Mack et al., 2014 [90]	Common mental disorder	Survey questions based on diagnostic criteria: DSM-IV	Inpatient mental health treatment, outpatient mental health treatment
Park et al., 2012 [100]	Major depressive disorder	Survey questions based on diagnostic criteria: DSM-IV	Any mental health professional, support groups, spiritual or religious advisors, healer
Pelletier et al., 2017 [104]	Anxiety disorder, major depressive disorder	Survey questions based on diagnostic criteria: DSM-IV	Any mental health professional, support groups
Reaume et al., 2021 [110]	Anxiety disorder, major depressive disorder, bipolar and substance use disorder	Survey questions based on diagnostic criteria: DSM-IV	Any mental health professional
Reavley et al., 2020 [111]	Anxiety disorders, depression, bipolar, any other disorder	Screening instrument/ scale: K6	Any mental health professional, hospitalization, support groups
Shafie et al., 2021 [119]	Anxiety disorders, mood disorders and alcohol abuse disorders	Survey questions based on diagnostic criteria: DSM-IV	Any mental health professional, support groups, spiritual or religious advisors, healer
Spinogati et al., 2015 [123]	Mood disorders, stress-related and somatoform disorders, other mental disorder	Clinical diagnosis: registry data	Hospitalization
Volkert et al., 2018 [128]	Anxiety disorder, depressive disorder, substance-related disorder	Survey questions based on diagnostic criteria: DSM-IV	Any mental health professional, hospitalization, support groups
Wang et al., 2019 [129]	Anxiety disorder, major depressive disorder	Survey questions based on diagnostic criteria: DSM-IV	Any mental health professional
Werlen et al., 2020 [132]	Anxiety disorder, depression, attention deficit hyperactivity disorder	Screening instrument/ scale: Generalized Anxiety Disorder 7, PHQ-9, and Adult ADHD Self-Report Scale Screener	Any mental health professional
Whitley et al., 2017 [134]	Anxiety disorders, mood disorders and alcohol abuse disorders	Screening instrument/ scale: K10	Any mental health professional, hospitalization, support groups
Yang et al., 2020 [139]	Major depressive disorder	Screening instrument/ scale: K6	Any mental health professional
Rim et al., 2023 [105]	Anxiety disorder, major depressive disorder, bipolar and substance use disorder	Survey questions based on diagnostic criteria: DSM-IV	Any mental health professional, support groups
Chaudhry et al., 2022 [23]	Different level of psychological distress	Screening instrument/ scale: K6	Any mental health professional, hospitalization
Reich et al., 2023 [103]	Substance use disorder, psychotic disorder, obsessive-compulsive disorder	Survey questions based on diagnostic criteria: DSM-IV	Inpatient mental health treatment, outpatient mental health treatment
Errazuriz et al., 2023 [40]	Anxiety disorder, major depressive disorder	Research diagnostic interview: ICD-10 diagnoses from administrative databases	Any mental health professional, support groups
Silvia et al., 2022 [120]	Anxiety disorders, mood disorders and alcohol abuse disorders	Survey questions based on diagnostic criteria: DSM-IV	Any mental health professional, spiritual or religious advisors, healer
Björkenstam et al., 2022 [10]	Anxiety disorder, depressive disorder, substance-related disorder	Research diagnostic interview: ICD-10 diagnoses from administrative databases	Inpatient mental health treatment, outpatient mental health treatment
Blasbalg et al., 2023 [13]	Anxiety disorder, depressive disorder, substance-related disorder, sleep disorders	Research diagnostic interview: ICD-10 diagnoses from administrative databases	Psychiatrist

**Table 4:** Study quality and outcomes

Author & published year	Quality Grade	Outcomes
Abramovich et al., 2020 [2]	Good quality	Transgender individuals had a higher number of mental health-related primary care visits. The total mean and standard deviation of mental health-related primary care physician visits were higher among transgender women compared with the transgender sample.
Caron et al., 2012 [21]	Fair Quality	Among the 406 participants who experienced at least one mental illness, 212 (52%) reported using mental healthcare services at least once in their lifetime, and these participants mainly experienced major episodes of depression (*N* = 129; 61%).
Chiu et al., 2018 [24]	Fair	White respondents were more likely to use mental health services, while respondents from China had the poorest self-rated mental health and unwilling to seek help.
Choi, Diana & Nathan, 2014 [26]	Good quality	Compared to 35–49 age group, participants aged 65 + had the lowest odds. Compared to non-Hispanic Blacks and Hispanics, non-Hispanic Whites were more likely to receive treatment. Participants with a college education and higher income were more likely to use MH treatment. Married and employed were less likely to received treatment.
Chong et al., 2012 [27]	Good quality	People with severe disability were more likely to seek help compared to those with moderate and mild symptoms. Women in Singapore were more likely to seek help from professionals in the mental health sector instead of people in the medical sector.
Chow & Mulder, 2017 [28]	Poor Quality	People aged between 20–39 were more likely to access mental health service. Māori have the highest rate of access, while Asians were less likely to accesse mental health services compared to other ethnic groups.
De Luca et al., 2015 [30]	Fair Quality	There was no difference mental health treatment utilization between veterans and non-veterans for non-Hispanic Whites. There was an overall lack of healthcare usage among racial/ ethnic minority non-veterans compared to veteran participants.
Durbin et al., 2015 [31]	Fair Quality	Immigrants from West and Central Africa were more likely to access primary care, while this was least common for immigrants from industrialized countries and East Asia and the Pacific. Long-term residents were more likely to use mental health services than all immigrant region groups.
Elbogen et al., 2013 [37]	Fair Quality	Women were more likely to report mental health problems and to obtain treatment. Women were more likely to report child care as a problem, while men wanting to solve mental health problems on their own and reluctant to seek help. Veterans with more severe PTSD or depression symptoms were more likely to access treatment.
Erlangse et al., 2017 [39]	Good quality	People bereaved by spousal suicide had an elevated risk of a mental disorder when compared with the general population. There was an increased use of psychiatric in-patient care among people bereaved by a spouse’s suicide, and 1 or more appointments with private psychiatrists or psychologists.
Fleury et al., 2012 [21]	Fair Quality	Middle-aged individuals were more likely to use services than those under 25 and those over 65. Individuals who view their mental health as poor were 73% more likely to use services. Female participants were 49% more likely to use services. Individuals living in neighbourhoods where renters outnumber homeowners used fewer health services.
Forslund et al., 2020 [43]	Good quality	Women were 1.5–2 times more likely than men to utilize mental health care in all age groups. Among adult women, mental health care utilization peaked in the 45–64 age group with a prevalence of 21.9% in 2017.
Gazibara et al., 2021 [45]	Good quality	For women, the peak effect was observed in the first 4–6 months after a sibling’s death, while for men the peak was observed in the first 3 months after the death. In general, there was increased contacts with the GP over 24 months before the death and over the first 3 months after the death.
Glasheen et al., 2019 [46]	Good quality	For participants with low/ moderate mental illness, mental health service use was less prevalent among adults with proximal residence transience compared with those with no transience. Cost, no transportation, inadequate service were the main barriers to service use.
Goncalves et al., 2014 [47]	Good quality	There were no significant differences in the use of healthcare services for mental health problems by education, place of residence or chronic physical health problems. There was an inverse relation between age and the likelihood of seeking help. Being female and single were more likely access MH services.
Harber-Aschan et al., 2019 [54]	Good quality	Mental health service use was more prevalent among participants reporting greater need, was more frequently observed in women, persons aged 50–59, and those of divorced/separated/widowed relationship status. People with relatively small social networks and more stressful life events were more likely to use mental health service. Mental health service did not vary by ethnicity, migrant status or education.
Huynh et al., 2016 [55]	Good quality	18 to 29: experiencing a higher number of stressful events were associated with mental health service use. 30 to 64: family history of mental disorder, perceiving neighborhood disorder was correlated with service use. However, having an active occupation, stigma was negatively associated with mental health service use.
Islam et al., 2017 [66]	Good quality	Women, middle-aged, white populations, unemployed, people who perceived their health as poor and those living in household food insecurity were associated with higher MH service use. While higher education levels were associated with lower MH service use.
Jacobi & Groß, 2014 [67]	Poor Quality	Twelve-month service utilization rates were lower in young adults and older adults. Middle-aged adults with mental disorders are more likely to access mental health services. Women with diagnosed mental disorders were 2.3 times more often to access health services compared to men (*p* < .001).
Kaul et al., 2017 [68]	Good quality	More cancer survivors reported having seen a mental health professional than the comparison group. Barriers: could not afford mental health care. Among survivors who reported not being able to afford mental health care, those with moderate and severe distress reported this affordability barrier more often than those without distress.
Lipson et al., 2019 [87]	Poor Quality	The most common location for college students to receive services was on campus, the percentage of perceived and personal stigma decreased over time. Among students with depression, there was no noticeable change in levels of perceived stigma over time, but personal stigma decreased from 8.2% to 5.1%.
Mack et al., 2014 [90]	Good quality	Women reported higher rate of lifetime service use than men in all age groups. Service use rates were particularly low among the elderly with mental disorders, of which 14.8% of women and 4.0% of men reported service use in the past year. Never married and not being employed were associate with higher service use.
Park et al., 2012 [100]	Fair Quality	Gender, area of residence, marital status, educational level, and type of occupation were not associated with MH service use. The main barrier to treatment was poor understanding of mental disorder, cultural issue, and non- recognition of depression and stigma.
Pelletier et al., 2017 [104]	Fair Quality	People with both generalized anxiety disorder and major depressive disorder were more likely to seek help (nearly 75%), while less than 60% of individuals with GAD consulted a health professional. There were no statistically significant outcome for age-related effects.
Reaume et al., 2021 [110]	Good quality	Having a physical health problem was associated with higher odds of using mental health care, the ORs were higher among individuals with physical health problems and comorbid mental health, or substance use problems.
Reavley et al., 2020 [111]	Fair Quality	Every one-unit increase in discrimination was associated with an increased visit to the hospital or specialist doctor. Concurrent experiences were more closely related to health service use than past experiences, while the past experience of discrimination was associated with a greater number of visits to hospitals or specialist doctors.
Shafie et al., 2021 [119]	Good quality	Participants from older age groups had lower odds of seeking help from any service providers compared to those aged 18–34 years, Females, divorced/separated, unempolyed were higher MH service use. Widowed was correlated with lower odds of seeking help.
Spinogati et al., 2015 [123]	Fair Quality	Females were more likely to access mental health services in most countries, except for North Africa. Compared to immigrants, natives have higher rates of service use. There are no significant differences in employment and retirement.
Volkert et al., 2018 [128]	Fair Quality	Males and higher education were associated with lower or no service use in the past 12 months. Geographical location also impacts on the likelihood of service use. The most frequently reported barriers to service utilization were personal beliefs, practical barriers, and stigma-related barriers.
Wang et al., 2019 [129]	Good quality	Being female, ages 35 to 49 years, White ethnicity, and living unhealthy were more likely to report mental health service use. The odds of service use were lower in people with insurance, and illicit drug or alcohol abuse was positively associated with increased mental health service use.
Werlen et al., 2020 [132]	Fair Quality	Participants with all three diagnoses (ADHD, anxiety and depression) had the highest service utilization. Females were more likely to access mental health services. Risky alcohol use was associated with lower current service utilization, while risky non-prescribed prescription drug use was associated with higher current service utilization.
Whitley et al., 2017 [134]	Fair Quality	Immigrants reported a lower frequency of healthcare services utilization but higher health services satisfaction scores than non-immigrants. Respondents born in Asia or Africa had fewer healthcare services utilization but higher service satisfaction scores.
Yang et al., 2020 [139]	Fair Quality	Asians were significantly less likely than whites to access mental health service use. Asians had greater access to mental health services when the need for treatment was recognized by the patient. For barriers to service use, Asians were less likely than whites to endorse cost as a treatment barrier, the major barrier for Asians is stigma and cultural factors.
Rim et al., 2023 [105]	Fair Quality	The overall treatment rate of mental disorders in Korea was lower than the global treatment rate. The top three reasons for not receiving treatment for a mental disorder were “I thought I could solve that problem by myself,” “I thought I didn’t have a mental disorder,” and “I thought the problem would improve on its own”. Barrier: Stigma
Chaudhry et al., 2022 [23]	Fair Quality	Individuals with moderate and serious psychological distress were more likely to have had a mental health visit. Two of the structural barriers were significantly associated with all mental healthcare utilization measures, which are “Told health care coverage not accepted” and “Delayed care, couldn’t get an appointment soon”.
Reich et al., 2023 [103]	Good quality	Female, participants with mental disorders in more than one diagnostic category were more likely to use MH services. Age, migration background was unrelated to health service in the present dat. High education were associated with less health service use. Barrier: stigma, social support and internal control beliefs.
Errazuriz et al., 2023 [40]	Fair Quality	Immigrants were less likely than non- immigrant to have accessed mental health services. There was a positive association between severity of symptoms and access to mental health services, and this association only observed among immigrants, but not among non-immigrants.
Silvia et al., 2022 [120]	Good quality	Single participants had lower odds of having received treatment than married patients. Participants with basic or secondary education had 58% lower odds of having received treatment than those with university levels. Attitudinal barriers were the most commonly reported barrier to treatment, followed by low perceived need and structural barriers.
Björkenstam et al., 2022 [10]	Good quality	Refugees had the lowest rate for overall psychiatric care utilization. In both non-refugee immigrants and refugees, those residing 10 years or more in Sweden had the highest hazard ratios for psychiatric care utilization.
Blasbalg et al., 2023 [13]	Good quality	Adults were reluctant to utilize medical health services during the pandemic. The association between age and receiving diagnoses and treatment were significant in both 2018 and 2019. There were no statistically significant impacts on mood disorders during the pandemic lockdown.

Twenty-seven studies (67.5%) analyzed the rate of mental health service use over the last 12 months, six studies (15.0%) focused on lifetime service use, and three studies (7.5%) assessed both 12-month and lifetime mental health service use. A few studies examined other time frames, with single studies investigating mental health service use over the past 3 months, 5 years, and 7 years, and one included study considered mental health service use during the 24 months before and after a sibling’s death.

Twenty of the forty studies were classified as good quality (50.0%), seventeen as fair (42.5%), and three as poor quality (7.5%).

### Overview of samples and factors investigated

The included studies examined a range of different factors associated with mental health service use. These included gender, age, marital status, ethnic groups, alcohol and drug abuse, education and income level, employment status, symptom severity, and residential location. The review identified service utilization factors related to socio-demographics, differences in utilization across countries, emerging socio-demographic factors and contexts, as well as structural and attitudinal barriers. These are described in further detail below.

### Socio-demographic characteristics

#### Gender

Fifteen studies analyzed the association between gender and mental health service use, with fourteen studies reporting that mental health service use was more frequent among females with mental disorders than males [2, 37, 42, 43, 47, 54, 66, 67, 90, 103, 119, 123, 128, 130]. A South Korean study concluded that gender was not associated with mental health service use [100], which might be due to the small sample size of 362 participants in the study.

#### Age

Fourteen studies investigated age in association with mental health service use. Nine studies concluded that mental health service use was lower among young and old adult groups, with middle-aged persons with a mental disorder being most likely to access treatment from a mental health professional [26, 42, 43, 47, 54, 66, 67, 123, 130]. Forslund et al. [43] reported that mental health service use for women in Sweden peaked in the 45-to-64-year age group, while amongst males, mental health service use was stable across the lifespan. In contrast, two articles from New Zealand and Singapore each reported that young adults were the age group most likely to access services [28, 119]. Reich et al. [103] concluded that age was unrelated to mental health service use when considered for the whole population, but sex-specific analyses reported that mental health service use was higher in older than younger females, while the opposite pattern was observed for males. A Canadian study using community health survey data also observed no significant age-related differences in mental health service use [104].

#### Marital status

There was mixed evidence concerning marital status. Studies from the United States and Germany concluded that participants who were married or cohabiting had lower rates of mental health service use [26, 90], while Silvia et al. [120] found that mental health service use was higher among married participants in Portugal. Shafie et al. [119] reported being widowed was associated with lower rates of mental health service use in Singapore.

#### Ethnic groups

Eight studies examined the relationship between ethnic background and mental healthcare service use. Non-Hispanic White respondents were more likely to use mental health services in Canada and the United States [24, 26, 30, 130, 139], while Asians showed lower rates of mental health service use [28, 139]. Chow & Mulder [28] investigated mental health service use among Asians, Europeans, Maori, and Pacific peoples in New Zealand. They concluded that Maori had the highest rate of mental health service use compared with other ethnic groups. De Luca et al. [30] reported that mental health service use was lower among ethnic minority non-veterans compared to veterans in the United States, especially for those with Black or Hispanic backgrounds. In contrast, a study conducted in the UK found that mental health service use did not vary by ethnicity, with no difference between white and non-white persons [54].

#### Alcohol and drug abuse

Two studies reported risky alcohol use was negatively associated with mental health service use [26, 132]. However, within the time frame of the current review, there was insufficient published evidence on the impact of drug abuse on mental health service use among people with mental disorders. Choi, Diana & Nathan [26] found that drug abuse can lead to lower rates of mental health service use in the United States. In contrast, Werlen et al. [132] reported that risky use of (non-prescribed) prescription medications was associated with higher rates of mental health service use in Switzerland.

#### Education, income, and employment status

Four studies analyzed the relationship between education level, income, and mental health service use. Higher levels of educational attainment [26, 120] and higher income [26] were generally reported to be associated with an increased likelihood of mental health service use. However, Reich et al. [103] observed that in Germany, high education and perceived middle or high social class were associated with reduced mental health service use. One paper reported no significant difference in mental health service use in South Korea, possibly due to the small number of people accessing mental healthcare services [100].

Three studies reported that compared to those who are unemployed, those in work were less likely to use mental health services [26, 90, 119]. This outcome aligned with a Canadian study consisting of immigrants and general populations, Islam et al. [66] concluded that immigrants who were currently unemployed had higher odds of seeking treatment than those who were employed. However, an Italian [123] and a South Korean study [100] found that employment status was not related to mental health service use.

#### Symptom severity

Ten studies investigated the association between symptom severity and mental health service use and ten papers concluded that participants with moderate or serious psychological symptoms were more likely to use mental health services compared to those with mild symptoms [23, 27, 66, 103, 120, 123, 130, 139]. Other studies showed that study participants who viewed their mental health as poor [42], who were diagnosed with more than one mental disorder [103], and those who recognized their own need for mental health treatment [54, 139] were more likely to receive mental health services.

#### Residential location

 Three studies investigated the association between residential location and mental health service use. Volkert et al. [128] concluded that the rates of mental health service use in Germany were significantly lower among those living in Canterbury than those living in Hamburg. A Canadian study found individuals living in neighborhoods where renters outnumber homeowners were less likely to access mental health services [42]. In the United States, for participants with low or moderate mental illness, mental health service use was lower for those residing closer to clinics [46].

### Immigrants & refugees

The reviewed research found that non-refugee immigrants had slightly higher rates of mental health service use than refugees [10]. Other research found that long-term residents were more likely to access services than immigrants regardless of their origin [31, 134]. For example, Italian citizens were found to have higher rates of mental health service use compared to immigrants, especially for affective disorders [123]. In Canada, immigrants from West and Central Africa were more likely to access mental health services compared to immigrants from East Asia and the Pacific [31]. Research from Chile found that the rates of mental health service use were similar for immigrants and non-immigrants [40]. Although, a positive association between the severity of symptoms and rates of mental health service use was only observed among immigrants [40]. Whitley et al. [134] found that immigrants born in Asia or Africa had lower rates of mental health service use, but higher rates of service satisfaction scores compared to immigrants from other countries.

### Emerging areas

Our literature review identified several areas in which only a small number of studies were found. We briefly describe them here as these may reflect emerging areas of research interest. Few published articles examined mental health treatment among participants with mental disorders together with chronic physical health conditions, and we only included the papers in this systematic review if they contained a healthy comparison group. We identified two papers that focused on survivors of adolescent and young adult cancer [68] and participants with physical health problems [110]. Both studies reported that participants with other chronic conditions reported higher rates of mental health service use than the general population [68, 110].

Two studies compared treatment seeking among people experiencing stressful life events. Erlangsen et al. [39] investigated the impact of spousal suicide, and Gazibara et al. [45] examined the effect of a sibling’s death on mental health service use. People bereaved by relatives’ deaths were more likely to use mental health services than the general population [39, 45]. The peak effect was observed in the first 3 months after the death for both genders, while evidence of an increase in mental health service use was evident up to 24 months before a sibling’s death and remained evident for at least 24 months after the death [45].

One paper studied the impact of the COVID-19 pandemic lockdown on mental health service use. An Israeli study concluded that compared to 2018 and 2019, adults reported they were reluctant to receive treatment during the pandemic lockdown and observed a decrease in mental health service use [13].

### Structural and attitudinal barriers

In addition to the research considering a range of population characteristics (e.g. male, younger, or older age), several papers examined how attitudinal and structural factors were associated with mental health service use. The most frequently reported of these factors were cost [23, 46, 68, 120], lack of transportation [46, 83], inadequate services/ lack of availability [23, 46, 83, 128], poor understanding of mental disorders and what services were available [10, 11, 22, 83, 100, 105, 120], language difficulties [10], and stigma-related barriers [83, 100, 103, 105, 128]. Cultural issues and personal beliefs may influence the understanding of mental disorders and prevent people from using mental health services due to mistrust or fear of treatment [100, 128]. The review also observed some unique barriers to different population groups. Choi, Diana & Nathan [26] mentioned that lack of readiness and treatment cost were the biggest difficulties for older adults, while young participants were more concerned about stigma. Females also reported childcare as a factor limiting their ability to use mental health services, while the evidence reviewed argued that males prefer to solve mental health issues on their own, with internal control beliefs and lack of social support likely reducing their use of mental health services [37, 103].

## Discussion

### Summary of evidence

This systematic review investigated mental health service use among people with mental disorders and identified the factors associated with service use in high-income countries.

Most studies found that females with mental health conditions were more likely to use mental health services than males. The relationship between age and mental health service use was bell-shaped, with middle-aged participants having higher rates of mental health service use than other age groups. Possible explanations included that the elderly might be reluctant to disclose mental health symptoms, they might attribute their mental health symptoms to increasing age [20], and they may prefer to self-manage instead of seeking help from health professionals [44]. Caucasian ethnicity and higher household income were also associated with higher rates of mental health service use. Greater use of mental health services was observed in participants with severe mental symptoms, including among veterans [19, 37, 92]. Two studies also concluded that compared to other cultural groups, Asian respondents were more likely to receive treatment when problems were severe or had disabling effects [86, 97]. There was mixed evidence regarding employment status, although some studies found employment to be negatively related to receiving treatment [26, 90], and unemployed people are more likely to seek help [119]. There was inconsistent evidence for the association between marital status and service utilization. This contradictory evidence on marital status might be attributed to a lack of specification, some papers categorize it as married and non-married [26, 71, 131], while others further differentiate between those who were widowed, separated, and divorced [90, 119].

### Immigrants

A number of studies showed that immigrants can face unique stressors owing to their experience of migration, which may exacerbate or be the source of their mental health issues, and impact the use of mental health services [1, 8]. These include separation from families, support networks, linguistic and cultural barriers [9, 113].

Due to the increased number of international migrants, immigrants’ mental health status and healthcare use has drawn growing attention [7, 77, 99]. Kirmayer et al. [70] and Helman [57] found that culture might be associated with people’s attitudes and understanding of mental health, influencing help-seeking behaviors. In general, the current results showed that immigrants and refugees were less likely to use mental health services than their native-born counterparts, and this finding was consistent with previous studies [75, 82, 127]. For immigrants, the length of stay in the host country was closely related to rates of mental health service use, which was argued to reflect increasing familiarity with the host culture and language proficiency [1, 59].

### Emerging areas

Both mental disorders and chronic diseases contribute significantly to the global burden of disease. Prior studies have shown that people with chronic disease have a higher chance of experiencing psychological distress [6, 14, 68, 73], and vice versa [49, 74]. Hendrie et al. [58] concluded that respondents with chronic diseases were more likely to attend mental healthcare and reported higher costs. Negative experiences and stressful consequences related to chronic disease might contribute to the increased potential for mental illness but more opportunities to seek help from health professionals [60, 108, 135]. People with chronic diseases and mental health problems might experience more long-term pain and limitations in their daily lives, and these stressors can exacerbate their health conditions, and impact their attitude toward seeking help.

The COVID-19 pandemic had a major impact on mental health service use worldwide, the hospital admission and consultation rate decreased dramatically during the first pandemic year [118]. This reduction in service access might be a side effect of social distancing measures taken as mitigation measures, reducing both inciting incidents and physical access to services.

Financial difficulty, service availability, and stigma were frequently identified in the literature as structural and attitudinal factors associated with lower rates of mental health service use. These factors were associated with the different rates of mental health service use for different ethnicities. For example, Asian people were less likely than other groups to identify cost as a factor limiting their use of mental health services, with a major barrier for Asian people being stigma and cultural factors [139].

### Limitations

This systematic review employed a broad search strategy with broad search terms to capture relevant articles. Rather than emphasizing a particular mental disorder, this review focused on the rates of mental health service use among adults aged 18 years or older who were experiencing a common mental disorder. However, this review still contained limitations. First was the potential for selection bias. Although we used various search terms for mental health service use and mental disorder, it is possible that the service use was not the primary research question for some papers, or that the relevant service use outcome was not statistically significant- in these cases, if the information was not reported in the abstract, relevant papers might have been missed. It is also important to note that this systematic review includes studies conducted in different countries and that the mental health systems and opportunities for access vary among countries. We only searched for full-text peer-reviewed articles published in English. Grey literature and papers published in other languages were excluded from the search. Most of the included literature used self-reported data to measure service access, and these data can be liable to recall bias. Studies using administrative data were also included in the systematic review, and we note that although they have large datasets, compared to survey data, there is often a lack of adequate control variables included to minimize possible confounding influences.

### Future research

There is a need for more published articles on several aspects that may influence the service utilization among people with mental disorders, including the impact of residential or neighborhood areas, and household income across various income groups. These aspects are important population characteristics that require further research to inform the targeting and type of support (e.g. low-cost, accessible). Additionally, there was a lack of longitudinal research on mental health service use, future studies could use the data to identify changes over time and relate events to specific exposures (e.g. Covid-19 pandemic). Future studies can investigate the cost of mental health treatment in detailed aspects, (e.g. publicly funded mental health services, community-based support for free or low-cost mental health services). Overall, there was a lack of studies for ethnic minorities, given ethnic minority groups were more vulnerable to mental disorders but with less mental health service use. Future research can expand gender identity representation in data collection and move beyond the binary genders. People with non-binary gender identities can face greater challenges and disadvantages in mental health and mental health service use.

## Conclusion

This review identified that middle-aged, female gender, Caucasian ethnicity, and severity of mental disorder symptoms were factors consistently associated with higher rates of mental health service use among people with a mental disorder. In comparison, the influence of employment and marital status on mental health service use was unclear due to the limited number of published studies and/ or mixed results. Financial difficulty, stigma, lack of transportation, and inadequate mental health services were the structural barriers most consistently identified as being associated with lower rates of mental health service use. Finally, ethnicity and immigrant status were also associated with differences in understanding of mental health (i.e. mental health literacy), effectiveness of mental health treatments, as well as language difficulties. The insights gained through this review on the factors associated with mental health service use can help clinicians and policymakers to identify and provide more targeted support for those least likely to access services, and this in turn may contribute to reducing inequalities in not only mental health service use but also the burden of mental disorders.

### Supplementary Information

Supplementary Material 1.

## Data Availability

All data and materials related to the study are available on request from the first author, yunqi.gao@anu.edu.au.
