# Supplementary material for: Examining the mental health services among people with mental disorders: a literature review
Source: BMC Psychiatry. 2024 Aug 20;24:568. doi: 10.1186/s12888-024-05965-z (PMC11334396; doi:10.1186/s12888-024-05965-z)
Supplement: Supplementary file 1 — Supplementary Material 1. [file 12888_2024_5965_MOESM1_ESM.docx]

**Supplementary Appendix**

Supplementary Table 1. Search strategy.

PubMed (Title, Abstract)

| # | Search terms |
| --- | --- |
| 1 | “mental disorder” OR “mental illness” |
| 2 | “mental health service use” OR “health service use” OR “health service utili*” OR “health care use” OR “health care utili*” |
| 3 | #1 AND #2 |
| Additional filters | ‘English language only’ |

Scopus (Title, Abstract, Keywords)

| # | Search terms |
| --- | --- |
| 1 | “mental disorder” OR “mental illness” |
| 2 | “mental health service use” OR “health service use” OR “health service utili*” OR “health care use” OR “health care utili*” |
| 3 | #1 And #2 |
| Additional filters | ‘English language only’ |

Web of Science (All fields)

| # | Search terms |
| --- | --- |
| 1 | “mental disorder” OR “mental illness” |
| 2 | “mental health service use” OR “health service use” OR “health service utili*” OR “health care use” OR “health care utili*” |
| 3 | #1 And #2 |
| Additional filters | ‘English language only’ |
